# Surgery of the War, 1861 to 18651President’s address, delivered before the Medical Society of the County of Erie, January 12, 1897.

**Published:** 1897-03

**Authors:** J. G. Thompson

**Affiliations:** Angola, N. Y.; Late Surgeon 77th Regiment, New York Volunteers


### BUFFALO MEDICAL JOURNAL.

Vol. XXXVI. MARCH, 1897. No. 8.

### Original Communications.

### SURGERY OF THE WAR, 1861 TO 1865.[1]

By J. G. THOMPSON, M. D., Angola, N. Y.,
Late Surgeon 77th Regiment, New York Volunteers.

PROGRESS in surgical science, during the war, was in the line of technique in operative procedures, improvement in surgical dressings, adaptations of dressings to the exigencies of individual cases, whether designed for immediate permanent location in hospitals or for transportation over rough roads for long distances to permanent hospitals, and in the organisation and conduct of hospitals, both field and general, and in the general care of the wounded.

The hustle and bustle incident to active campaigning, and the scarcity of medical books and periodicals and of appliances for scientific research, were adverse to the rapid development of new theories and facts, so characteristic of recent investigations.

The great number of autopsies, however, gave opportunity for the study of pathology, of which the Medical and Surgical History of the War gives evidence.

The personnel of the volunteer surgeons, and especially of the assistant surgeons, composed largely of recent graduates, might not have been quite up to the highest standard of proficiency, and it gives cause for regret that those brave soldiers, who so patriotically volunteered to defend our sacred institutions, should have become the victims ofttimes of inexperienced surgical work.

I have to congratulate myself, however, that the victim of my first amputation is now an honored citizen of Saratoga, and has been twice elected to the Assembly from that district.

During the progress of the war, system in operative surgery in the field and hospital arrangements were perfected, and the operations were principally done by skilled surgeons with competent assistants, selected by the chief surgeons of divisions from those of

1. President's address, delivered before the Medical Society of the County of Erie, January 12, 1897.

regimental officers, whose ability to do the work properly he could vouch for. The operating staff was under his constant supervision, and any proving incompetent were returned to their regiments and others assigned to their places.

The work in general hospitals was done principally by regular surgeons, United States volunteer surgeons and assistant surgeons, commissioned by the President after having been confirmed by the Senate, having no command, but under the orders of the surgeon-general, with rank and pay of regimental surgeons and assistants respectively. These were, many of them, of high professional standing, and made records worthy of themselves.

The greater part of the reports embodied in the surgical history of the war was written by these surgeons, the medical officers in the field having little time or opportunity to make full reports; besides they were not able to continue observation of their patients, they being removed to general hospitals as soon as the exigencies of the service required it.

Amputations were the most frequent capital operations done, 29,980 being recorded; and in the technique of this class of operations, army surgeons were in the advance. The flap operation was the most commonly used and the circular next. Several modifications of these methods were proposed and quite extensively performed.

In the Surgical History of the War, Vol. III., page 723, in one of my reported cases, I partially describe a modification of the ordinary flap operation, which I always considered original with myself, and which I used commonly. Later I find it mentioned in Agnew's Surgery, Vol. II., page 305, as his method. It is done by cutting down to the deep fascia with the scalpel, making the outlines of the flaps, dissecting back those divided tissues one-quarter to one-third the way, evenly, from the angles to the extremities of the flaps, and with the catling cutting the anterior muscular flap from without inward, and the posterior from within outward, each to the line of dissection. A circular sweep of the catling, and peeling back the periosteum one-half inch, prepares the way for the retractor and saw. This I consider an ideal flap operation, avoiding the redundancy of muscles, cutting the vessels more squarely across, and giving better margins for perfect coaptation of the flaps.

It was customary with many surgeons, when time permitted, to leave the cut surfaces exposed to the air for twenty minutes or more, to become seared or glazed over, before approximating them, experience teaching that this gave greater percentage of primary unions and less secondary hemorrhage. This taught us that we could not be too careful in securing all bleeding points before closing wounds in which we desired union by first intention. We infer also from this experience that the great carrier of infection is not common air.

Water dressings were almost universally used, because all experience up to that time was vastly in their favor. I am happy to say, however, that occasionally surgeons observed that wounds did well with dry dressings, when water was not obtainable, despite the lack of aseptic methods and antiseptic paraphernalia. Antiseptics were hardly known and little used, except chlorine and charcoal, and perhaps permanganate of potassa, for cleansing foul ulcers and suppurating and gangrenous wounds, with copperas and chloride of lime for disinfecting purposes. Few large wounds healed without pus formations. Exfoliation of bone, secondary hemorrhage, pyemia, septicemia and reamputations were of frequent occurrence. Surgical fever was expected after all capital operations. Its degree was not recorded, because clinical thermometers were not a part of the surgeon's armamentarium at that time.

Animal, sterilised and antiseptic ligatures were not in use, silk being almost universally adopted for ligatures and sutures. Ligatures were left long and brought out at the lower angle of the wound, knots being tied in those securing large vessels, to distinguish them in removal, which was done from the third to the tenth day, by pulling or twisting. Large wounds were frequently closed with adhesive plaster. While a prisoner in the Wilderness, without a supply of silk, I used linen and cotton thread for ligatures and sutures, and wounds did fairly well with them.

The worst enemy we had to fight while prisoners was the maggot. These insects would unavoidably get into the wounds, and soon you would discover a purple line, an inch or more from the margin of the flaps. No time was then to be lost in opening up the wounds, dropping in a little chloroform, covering all for a short time with the hands, after which a dash of water dislodged every vestige of the colony. Nothing else being available for that purpose, the mother of invention directed chloroform, and its action was perfect.

Most of the fractures were compound and comminuted, and required firm dressings for transportation in ambulances, and often in army wagons. Plaster-of-paris dressings subserved the purpose well, and were extensively used at field hospitals. Wire splints—notably Smith's anterior—were much in use for suspending the limb and as a firm support in transportation.

The invaluable cracker box was one of the institutions of the army, useful for splints as well as for tables, seats and other tent furniture. It was also used to mark the resting-place of our fallen comrades, who were not embalmed and sent home for interment. In the general hospitals all the valuable devices and appliances known to surgical science at that time were furnished by the government, and generally used.

Sterilisation of instruments, sponges and dressings was not thought of, and the germ theory was not born. Septicemia was recognised, but that the cause of many, if not all, septic infections may be traced to bacteria was hardly "dreamed of in our philosophy." Admitting the germ theory to be the Alpha of etiological science, the alphabet is long; when shall we reach the Omega? It was considered better surgery, when hemorrhage from a wound demanded it, to open the wound and ligate the offending vessel, than to go above and ligate the main trunk, and experience taught that shot wounds often did better when laid freely open. Wounds from conical balls were most frequent and most severe, from the fact that their course is direct and they destroy everything in their track, while round balls are often deflected by fasciæ, and do not penetrate so deeply.

Excision of bones, during the first years of the war, was done quite extensively, the theory of surgeons being to save all the limbs possible. This theory, and consequently the practice, was materially modified as the war progressed, and more discretion was used in selection of cases for resection. The fact became pretty well established that excisions of bones of the upper extremities were attended with good results, giving useful limbs, while those of the lower extremity were not so successful, many of them resulting in nonunion, great shortening of the limb, and frequently followed by secondary amputations. A few selected cases did well when only a small section of the bone was removed, but extensive resection of the bones of the leg or thigh, and especially of the knee-joint, proved disastrous.

With the exception of the elbow, excision of joints showed a greater mortality than resection of bones immediately above the joints. In the upper extremities the percentage of excisions in gunshot fractures was 11.6 per cent.; in the lower extremities, 3 per cent. The fatality of excisions, in the whole series of cases reported, exceeds that of amputations 1.7 per cent.

Thirty cases of plastic operations were reported, with material improvement in most of them. No skin grafting cases were reported. Hermetical sealing of chest wounds was a procedure advocated by Assistant Surgeon Benjamin Howard, U. S. A., who was given extensive opportunities to test its merits, but without the success which the advocates of the operation had hoped. The external wound was dissected clean, and its edges approximated and sealed with lint and collodion. Invasion of the abdominal cavity, for the reparation of gunshot wounds of the contained viscera, was rarely practised, the opium treatment, pushed to the fullest tolerance, being the custom.

Anesthetics were used in 80,000 instances. The latest estimate of the number of cases in which chloroform was used gives 76.2 per cent. of the total number; ether, 14.7 per cent. ; and a mixture of chloroform and ether, 9.1 per cent. Total deaths reported from chloroform, 37 ; from ether, 4 ; chloroform and ether mixed, 2. These statistics and estimates give the greatest proportionate percentage of deaths from chloroform, with ether next, and chloroform and ether mixed least.

It was the practice of most surgeons to save as much of the limb as possible. Statistics prove that this was not the best practice. Amputations through the upper third of the leg, as reported, show a fatality of 27 per cent. ; of the middle third, 20.6 per cent.; and in the lower third, 27.6 per cent. Amputations through the middle third of the thigh, arm and forearm also show less fatality than through the upper and lower third. Primary amputations gave best results, those performed after thirty days next, and intermediary, or those performed during the inflammatory stage, gave far the greatest fatality.

To the Surgical History of the War, prepared under the direction of the surgeon-general by eminent army surgeons, I am indebted for the statistics and many of the conclusions summarised in this paper.

The three volumes of Surgical History of the War, together with the three Medical, should be in every physician's library. This history is valuable, not only for the vast amount of medical and surgical statistics, and the detailed experience of many of the most eminent surgeons of that time, but as a memorial to the brave patriots who dared death for the cause they loved, teaching those who were not participants in the bloody conflict the lesson of the terrible carnage of the war.

As the supply of published editions is exhausted, I would recommend to this society, which takes the initiative in many good works, that a committee be appointed to draft a suitable memorial to Congress, to be presented through our representatives, for the publication by the government of another edition of this valuable work, which shall be ample for all physicians who desire it, and that we use our best endeavors, through our delegates to state and national societies, to secure their coöperation for the accomplishment of this purpose.

